# Population-wide impact of a pragmatic program to identify and manage individuals at high-risk of cardiovascular disease: a cluster randomized trial in 120 villages from Northern China

**DOI:** 10.3389/fcvm.2024.1372298

**Published:** 2024-05-24

**Authors:** Siyu Chen, Lijing L. Yan, Xiangxian Feng, Jianxin Zhang, Yuhong Zhang, Ruijuan Zhang, Bo Zhou, Yangfeng Wu

**Affiliations:** ^1^ First Hospital, Peking University, Beijing, China; ^2^ The George Institute for Global Health, Peking University Health Science Center, Beijing, China; ^3^ Global Health Research Center, Duke Kunshan University, Kunshan, China; ^4^ Department of Preventive Medicine, Changzhi Medical College, Changzhi, China; ^5^ Hebei Provincial Center for Disease Control and Prevention, Shijiazhuang, China; ^6^ School of Public Health and Management, Ningxia Medical University, Yinchuan, China; ^7^ Department of Public Health, Xi'an Jiaotong University, Xi'an, China; ^8^ Department of Clinical Epidemiology and Evidence-Based Medicine, First Hospital, China Medical University, Shenyang, China; ^9^ Clinical Research Institute, Peking University Health Science Center, Beijing, China; ^10^ School of Public Health, Peking University Health Science Center, Beijing, China

**Keywords:** cardiovascular diseases, blood pressure, hypertension, primary health care, health policy

## Abstract

**Objectives:**

To explore the population-wide impacts of an evidence-based high-risk strategy for prevention of cardiovascular diseases in resource-poor populations.

**Methods:**

A cluster randomized controlled trial was conducted among 120 villages in rural China, with 60 on intervention and 60 on usual care as controls, for 2 years. The intervention emphasized training village doctors to identify high-risk individuals and administering standardized treatments focusing on hypertension management. A random sample of 20 men aged ≥50 years and 20 women aged ≥60 years was drawn from each village before randomization for the baseline survey, and another independent random sample with the same age and sex distribution was drawn at 2 years for the post-intervention survey. The primary outcome was the population mean systolic blood pressure (SBP). Secondary outcomes included the proportions of patients who received regular primary care, antihypertensive medications, aspirin, or lifestyle advice.

**Results:**

A total of 5,654 high cardiovascular risk individuals were identified and managed by village doctors in intervention villages for 15 months on average, with mean SBP lowered by 19.8 mmHg and the proportion with blood pressure under control increased from 22.1% to 72.7%. The primary analysis of the two independent samples (5,050 and 4,887 participants each) showed that population-wide mean SBP in intervention villages did not differ from that in control villages at 2 years (mean difference = 1.0 mmHg, 95% CI: −2.19, 4.26; *P* = 0.528), though almost all secondary outcomes concerning primary care indicators significantly increased in intervention villages.

**Conclusions:**

In our study, the pragmatic cardiovascular risk management program targeting on high-risk individuals significantly improved the quality of primary care. However, its impact on population blood pressure level and the burden of hypertension-related diseases appeared very limited.

**Clinical Trial Registration:**

ClinicalTrial.gov identifier, NCT01259700.

## 1 Introduction

Cardiovascular disease (CVD) is the leading cause of premature death and a major cause of disability worldwide (1, 2). There are two types of strategies for the prevention and control of CVD: high-risk strategies and population-based strategies. High-risk strategies focus on identifying and managing individuals at high risk of developing cardiovascular events such as myocardial infarction and stroke, for example, managing patients with hypertension. High-risk strategies are more attractive because the nature of “diagnosis and treatment” is more familiar to both the medical community and the public. The use of these strategies is more rational, and it is easier to obtain evidence from high-risk individuals. In fact, much more evidence from randomized controlled studies has been obtained for high-risk strategies (3, 4). As early as 2002, training primary care providers with tailored guidelines on the management of hypertension was proposed, and these providers were shown to be capable of significantly increasing the blood pressure control rate among targeted patients with hypertension (5). Recently, a large-scale cluster-randomized trial in China demonstrated the efficacy of antihypertensive treatment in patients with hypertension for reducing the incidence of conditions such as stroke and myocardial infarction (6). Based on this evidence from typical randomized trials of targeted high-risk individuals, most clinical guidelines recommend risk-based screening and management strategies, which have become the focus of public health policy in several countries, including China (7, 8). However, the impact of such high-risk strategies on population disease burden has long been a matter of debate because the majority of CVD cases are not developed from high-risk individuals (4, 9, 10). This important question is strongly associated with policy-making regarding the number one killer in the world but has not been evaluated in appropriately controlled studies. Previous trials assessing the effect of high-risk strategies for CVD prevention commonly recruited only the target population and followed them throughout the trials.

The China Rural Health Initiative (CRHI) study was designed to address this question with a rigorous randomized design, specifically to assess the potential population-wide impact of a pragmatic low-cost primary care program designed to improve the management of individuals with high cardiovascular risk by training primary care providers in rural China with a package of care focusing on blood pressure reduction (11). The present paper is to report the main results from the study.

## 2 Methods

**Figure 1:**
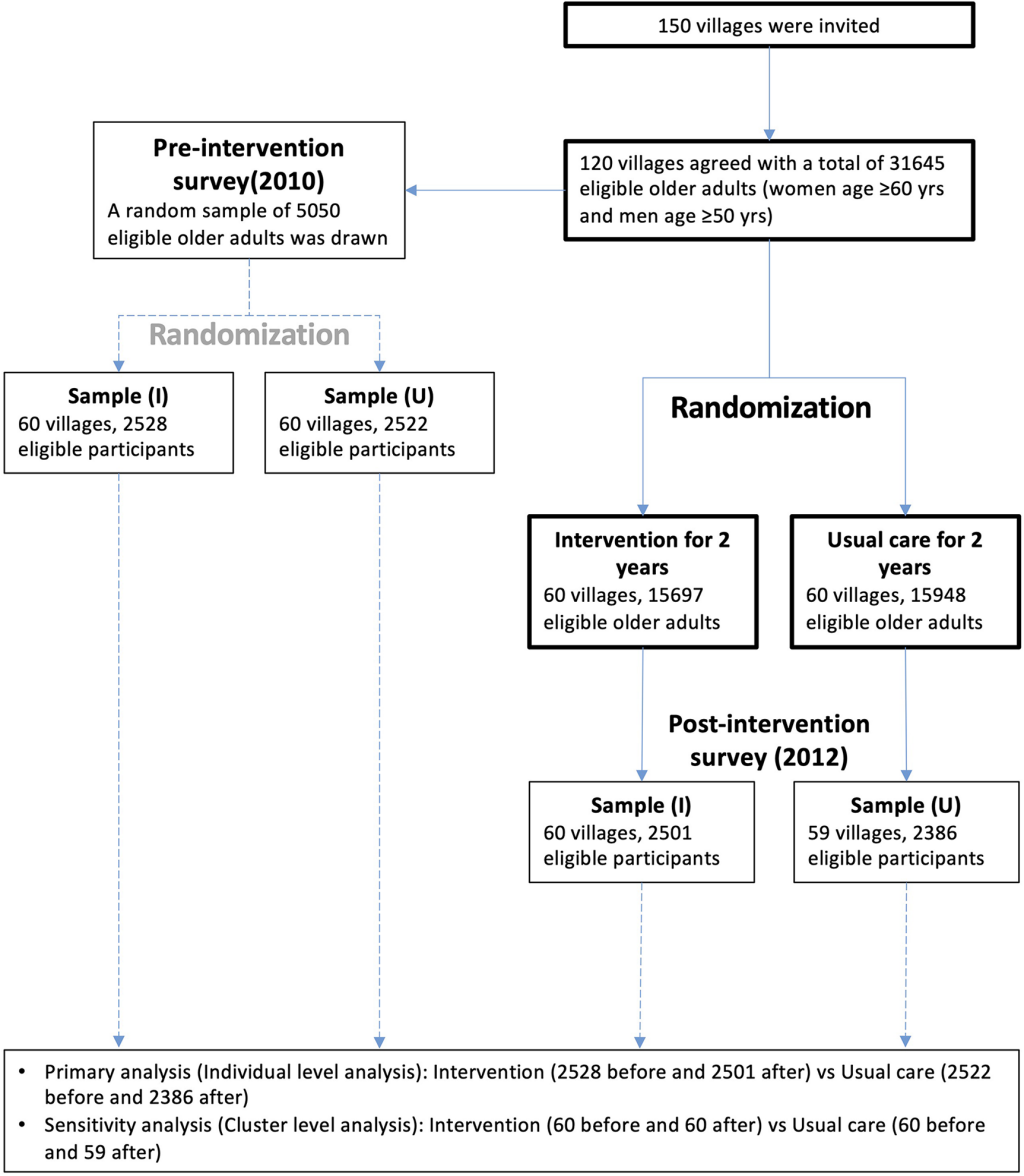
Trial flow chart.

The CRHI study used an innovative cluster-randomized trial in which 120 villages were recruited from 10 counties in 5 provinces in northern China. The villages were randomized at a 1:1 ratio into intervention and control groups stratified by county, with independent cross-sectional random samples drawn from each village (cluster) before and after the intervention (Figure 1). Primary care providers in the intervention villages received training to identify and manage individuals at high risk of CVD (for details, see below), and primary care providers in the control villages received no training. The CRHI study was registered at ClinicalTrials.gov (NCT01259700). Details of the CRHI study design have been published previously (11). The CRHI study was reviewed and approved by the Peking University Institutional Review Board in China and the Duke University Health System in the U.S. Cluster-level consent was obtained through a consultation process involving relevant provincial, county, regional and village authorities. Written informed consent was sought from all individuals selected for participation in the population surveys.

### 2.1 Setting

The CRHI study was conducted in five northern provinces and autonomous regions in China (Liaoning, Hebei, Shanxi, Shaanxi, and Ningxia) between September 2010 and November 2012. We purposefully selected two counties from each province and 12 towns, each comprising 10–30 villages, from each county. The counties were chosen to represent the overall level of socioeconomic development of the province. Within each of the 120 towns, eligible villages had a total population between 1,000 and 2,500. Ineligible villages were those in which regional government offices and regional healthcare centers were located. To reduce the risk of village-to-village contamination, we selected a single centrally located village from each town.

### 2.2 Randomization

We used a cluster-randomized, parallel group design. The subregion served as the unit of randomization, with assignment to the intervention or control arms in a 1:1 ratio using a central computerized process. Randomization was stratified by county in an effort to minimize confounding due to county-level factors such as different levels of economic development. Information about allocation status was divulged only after the collection of baseline data was completed.

### 2.3 Intervention

The CRHI intervention program was developed from evidence-based Chinese guidelines for the management of hypertension and CVD risk (12). The intervention program was designed to be affordable and pragmatic for implementation by village doctors. The objective was to increase the identification of individuals at high risk of CVD and improve their management. Village doctors were trained in the delivery of the intervention program by county physicians who had previously attended a two-day training course centrally provided by the study. Village doctors were trained in two one-day structured sessions conducted prior to initiation of the intervention and one month later. The duration of the intervention program was 2 years. Villages randomized to the control group received no intervention beyond what was generally available to all villages by county health authorities.

The CRHI intervention included a screening component focused on the identification of individuals at high cardiovascular risk, defined as an estimated 10-year absolute risk of stroke, myocardial infarction or cardiovascular death of at least 10% (13, 14). Specifically, an individual was defined as being at high risk if he or she met any of the following criteria: (1) a man or a woman with a prior diagnosis of coronary heart disease or stroke by a physician or (2) a man aged 50 years or older with a prior diagnosis of diabetes or a measured systolic blood pressure (SBP) ≥160 mmHg or (3) a woman aged 60 years or older with a prior diagnosis of diabetes or a measured SBP ≥160 mmHg. Serum lipids and blood sugar were not considered because there was a lack of laboratory devices for the measurements at China rural primary care system then. Smoking was not considered due to the large disparity in smoking rate between men and women in China, where only less than 3% of women smoke and hence the impact of smoking on the cardiovascular risk should have been included in the consideration on sex (15).

The CRHI intervention also included a standardized cardiovascular risk management package for high-risk individuals involving monthly follow-up at the village clinic with blood pressure measurements, advice about relevant aspects of lifestyle (with particular emphasis on smoking cessation and dietary salt reduction), drug treatment with antihypertensive drugs for those with an SBP ≥140 mmHg and antiplatelet therapy with low-dose aspirin (75–100 mg/day) for those without contraindications (such as prior cerebral or major noncerebral bleeding). The aspirin use was initiated only after SBP was lowered to below 140 mmHg. After beginning the intervention, when SBP was observed to be above 140 mmHg at any follow-up visit, an initial treatment with anti-hypertension medications or the addition of another medication was recommended.

In the intervention villages, each patient visit was documented by village doctors using a standard case management record (CMR). These records were collected at regular intervals by study staff and entered into a secure database from which reports on key performance indicators were generated. In addition, performance was monitored by phone calls with village doctors, monthly clinic visits, and six monthly home visits to a 5% random sample of patients under management. Modest performance-based financial incentive payments administered by local health authorities were made to village doctors on the basis of key performance indicators. The average annual payment was 1,396 RMB (equivalent to US$228 at the time of the study), which approximated one month's income for village doctors at the time of the study.

### 2.4 Outcome assessment

Systolic blood pressure (SBP) was the primary outcome. The secondary outcomes were the number of individuals who reported receiving the following: (1) regular clinical follow-up (defined as monthly visits to the village doctor); (2) advice about lifestyle changes (salt reduction and smoking cessation); (3) treatment with an antihypertensive drug; and (4) treatment with aspirin.

To objectively assess the effectiveness of the CRHI primary care program from a population health perspective, rather than from a clinical perspective, primary and secondary outcomes were assessed by independent study personnel before the implementation of the intervention (pre-intervention) and after the intervention (post-intervention) via surveys of separate random samples of residents in all 120 villages. A random sample of approximately 20 men aged ≥50 years and 20 women aged ≥60 years from each village was invited to participate in the pre-intervention survey, and an independent random sample of 40 adults was invited to participate in the post-intervention survey. Stratified sampling was used to ensure that each sample contained equal numbers of men and women.

In these surveys, blood pressure, weight, height and heart rate were measured and recorded by study personnel trained in the use of standardized protocols. Blood pressure was measured twice (with at least 3 min between measurements) using an automated electronic sphygmomanometer certified by the European Society of Hypertension International Protocol (Omron Intellisense HEM 7301 IT). A brief standardized questionnaire administered by trained interviewers collected information on disease history, lifestyle, medication use, and health care seeking behaviors.

### 2.5 Statistical methods

The study was designed to provide at least 80% power (two-sided alpha = 0.05) to detect a 1.6 mmHg pre- to post-intervention reduction in the mean SBP in the intervention villages compared to the control (11), with a sample size of 40 individuals from each village in each of the 2 surveys across the 120 clusters. If maintained long term, such a reduction would be expected to prevent at least one stroke among every 12 that would otherwise have occurred (16).

In the analysis of the primary outcome, SBP, individual-level data were used, and a generalized estimating equation extension of the linear regression model with an exchangeable covariance structure was used to account for clustering (17, 18). The intention-to-treat principle was followed.

All the secondary outcomes, which were binary variables, were analyzed following a strategy similar to that of the primary outcome using logistic regression with an exchangeable covariance structure to account for clustering.

Cluster-level analysis was performed as sensitivity analysis.

## 3 Results

The study flow chart is shown in Figure 1. Of the 150 villages invited to participate in the study, 120 agreed and were randomized. One village in control was withdrawn after randomization as a consequence of the village's incorporation within an adjacent urban area. The data were obtained from a total of 5,050 adults in the baseline survey (pre-intervention survey) and 4,887 in the post-intervention survey.

### 3.1 Characteristics of participants

The baseline and post-intervention samples were quite comparable in terms of age, sex and years of schooling. Mean age was about 66 years, half were women and about one-third were at high cardiovascular risk according to the study's definition. There were no significant differences between intervention and control villages in characteristics of participants at both baseline and post-intervention surveys. The characteristics were also comparable between the baseline and post-intervention surveys in terms of demographic characteristics (Table 1).

**Table 1:** Selected characteristics of participants at baseline and participants at post-intervention.

Characteristics (mean, SD or %)	Baseline		Post intervention	
	Intervention	Control	Intervention	Control
N	2,528	2,522	2,501	2,386
Age, years, mean (SD)	65.7 (8.20)	65.7 (8.08)	65.8 (7.79)	65.3 (7.64)
Female, *n* (%)	1,261 (49.9%)	1,260 (50.0%)	1,271 (50.8%)	1,211 (50.8%)
Years of schooling, mean (SD)	4.2 (3.62)	4.2 (3.55)	4.4 (3.54)	4.4 (3.59)
Current smoking, *n* (%)	773 (30.6%)	781 (31.0%)	721 (28.8%)	701 (29.4%)
Body mass index, mean (SD), kg/m^2^	24.1 (3.66)	24.2 (3.50)	24.4 (3.66)	24.2 (3.60)
Systolic blood pressure, mean (SD), mmHg	147.1 (23.97)	146.9 (24.58)	148.1 (22.54)	146.9 (22.80)
Diastolic blood pressure, mean (SD), mmHg	85.0 (13.82)	85.3 (14.58)	86.4 (13.96)	86.1 (13.84)
High cardiovascular risk, *n* (%)	897 (35.5%)	835 (33.1%)	971 (38.8%)	890 (37.3%)
Systolic blood pressure ≥160 mmHg, *n* (%)	706 (27.9%)	693 (27.5%)	700 (28.0%)	594 (24.9%)
Diabetes, *n* (%)	90 (3.6%)	74 (2.9%)	147 (5.9%)	126 (5.3%)
Stroke, *n* (%)	178 (7.0%)	164 (6.5%)	202 (8.1%)	189 (7.9%)
Coronary heart disease, *n* (%)	215 (8.5%)	188 (7.5%)	301 (12.0%)	303 (12.7%)
ICC for the primary outcome	0.06	0.05	0.07	0.03

### 3.2 Implementation of the intervention

Over the course of the two-year program, 85 village doctors in the 60 intervention villages identified a total of 5,654 patients who met the criteria for high cardiovascular risk. Details of the key performance indicators for the implementation of the intervention among these high-risk individuals over the two years of the study are shown in Table 2. The average duration of patient follow-up was 15 months, and a total of 86,507 clinic visits were completed. From the initiation visit to the last follow-up, the mean systolic and diastolic blood pressure decreased by 19.8 mmHg and 9.6 mmHg, respectively; hence, the proportion of patients with controlled blood pressure increased by 50.6%. Moreover, the proportion of individuals taking antihypertensive medications increased by 16.3%, the proportion of individuals taking aspirin increased by 42.4%, the proportion of individuals reducing salt intake increased by 42.5%, and 60.1% of smokers quit smoking (Table 2).

**Table 2:** Key indicators of the performance of village doctors in implementing the intervention program.

Key indicators	Initial visit	Last visit	Difference from initiation to last visit
Number attending	5,654	5,654	5,654
Mean SBP, mmHg (SD)	155.0 (20.71)	135.2 (10.52)	−19.8 (19.77)
Mean DBP, mmHg (SD)	90.3 (12.54)	80.7 (7.69)	−9.6 (12.44)
Blood pressure under control, %	22.1	72.7	50.6
Blood pressure under control among individuals with hypertension, %	25.6	83.1	57.5
Antihypertensive treatment, %	52.4	68.7	16.3
Antihypertensive treatment among individuals with hypertension, %	60.8	78.4	17.6
Taking aspirin, %	22.6	65.0	42.4
Reducing dietary salt intake, %	50.9	93.4	42.5
Smoking cessation among smokers, %	0.0	60.1	60.1

### 3.3 Effect on the primary outcome

The primary analysis showed no difference in the mean change in SBP from pre-intervention to post-intervention between the intervention and control villages (regression coefficient of the interaction term of intervention by visit: 1.0 mmHg; 95% confidence interval: −2.19–4.26 mmHg; *p* = 0.528). The sensitivity analysis at the cluster level showed that the net difference was 0.8 mmHg (*p* = 0.496) (Table 3).

**Table 3:** Effects on primary and secondary outcomes.

	Intervention			Control				*P* values for difference in change in 2 years	
Outcome	Pre-intervention (year 0)	Post-intervention (year 2)	Change in 2 years	Pre-intervention (year 0)	Post-intervention (year 2)	Change in 2 years	Net difference	Primary analysis	Sensitivity analysis
Primary outcome									
Systolic blood pressure	147.1 (145.34, 148.77)	148.1 (146.37, 149.80)	1.0 (−0.25, 2.31)	146.9 (145.31, 148.56)	146.9 (145.53, 148.24)	−0.1 (−1.12, 1.03)	1.0 (−2.19, 4.26)	0.528	0.496
Secondary outcomes									
Receiving regular primary care, %	7.4 (5.59, 9.13)	26.8 (23.48, 30.18)	19.5 (15.70, 23.24)	8.1 (6.45, 9.82)	17.7 (14.60, 20.82)	9.6 (6.10, 13.05)	9.9 (2.16, 19.39)	0.004	<0.001
Being treated with blood pressure lowing drugs, %	23.4 (20.67, 26.18)	34.0 (30.85, 37.20)	10.6 (6.34, 14.86)	25.3 (22.27, 28.29)	27.9 (25.22, 30.60)	2.6 (−1.50, 6.75)	7.9 (1.64, 14.88)	0.011	<0.001
Being treated with aspirin, %	3.8 (2.18, 5.42)	12.1 (9.27, 14.96)	8.3 (4.97, 11.66)	4.0 (2.86, 5.24)	5.6 (3.19, 8.06)	1.6 (−1.11, 4.26)	6.8 (2.66, 12.56)	<0.001	<0.001
Receiving advice on salt intake, %	9.1 (6.28, 12.00)	30.1 (25.49, 34.64)	20.9 (15.43, 26.42)	9.8 (6.57, 13.11)	21.6 (16.78, 26.44)	11.8 (5.86, 17.68)	9.2 (1.97, 17.74)	0.005	<0.001
Receiving advice on smoking cessation among smokers, %	7.0 (3.54, 10.43)	19.1 (12.43, 25.85)	12.2 (4.63, 19.68)	8.0 (4.45, 11.45)	14.3 (7.08, 21.49)	6.3 (−1.56, 14.23)	6.0 (−1.34, 16.40)	0.078	0.033

### 3.4 Effects on secondary outcomes

Over the 2-year intervention period, compared to the control group, the intervention group had significant greater increases in proportions receiving regular clinic follow-up (19.5% vs. 9.6%, *p* = 0.004), antihypertensive medications (10.6% vs. 2.6%, *p* = 0.011), aspirin (8.3% vs. 1.6%, *p* < 0.001), advice on salt intake reduction (20.9% vs. 11.8%, *p* = 0.005). The intervention group also had a higher proportion of smokers receiving advice on smoking cessation (12.2% vs. 6.3%, *p* = 0.078), though not significant. The sensitivity cluster level analysis showed similar results (Table 3).

## 4 Discussion

The CHRI was the first randomized trial to evaluate the population-wide effectiveness of a high-risk strategy for the prevention and control of CVD—proactivity in screening and managing individuals with high cardiovascular risk—for primary care providers in rural villages of northern China. The study, conducted in 120 rural villages, demonstrated that a pragmatic primary care program targeting individuals at high risk of CVD did not lower the population mean SBP, although it produced a 10% greater increase in receiving regular primary care, a 9% greater increase in receiving advice on salt reduction, an 8% greater increase in the use of antihypertensive treatment, and a 7% greater increase in the use of aspirin among older adults. These findings raise questions about the impact of strategies that focus only on high-risk patients on the population-wide disease burden.

Why did the CRHI PCP program yield an improvement in primary care but fail to result in a substantial reduction in the mean population blood pressure level? The population-wide impact of a high-risk strategy depends upon three main factors: first, the proportion of the targeted high-risk group in the whole population; second, the efficacy of the intervention strategy in identifying those at high risk and reducing the clinical outcomes among those identified at high risk; and third, the use of the primary care system to cover all those at high risk. In the present study, only one-third of the study participants could be defined as “high-risk individuals” according to the study definition. In addition, despite the intervention substantially increasing the number of high-risk individuals receiving regular primary care from village doctors, more than half of the high-risk individuals in the community still did not receive this service. Only a third of the population was at high risk, and approximately 50% of them were receiving regular primary care, while the observed 19.8 mmHg reduction in SBP among individuals managed by village doctors in the intervention villages may indeed be valid. The effect observed among the whole population should be less than 33% of the 50% reduction in 19.8 mmHg, equating to less than 3.3 mmHg.

Furthermore, there might be significant contamination of the CRHI primary care providers’ intervention program. For instance, data from control villages showed that all secondary outcomes improved, ranging from 1.6% for aspirin use to 11.8% for receiving advice on salt intake. Why the cluster randomization design failed to prevent contamination bias is not entirely known; however, the village doctors in both the intervention and control villages belong to the same public health systems that are centrally managed by the same county health authorities, who are responsible for the capacity building, duty responsibilities and performance appraisals of the village doctors. Considering the presence of contaminating factors such as regular primary care and advice on salt intake reduction, we posit that contamination bias may have mitigated approximately 50% of the intervention effect. Consequently, the effect of the CRHI-related PCP intervention at the population level should be further reduced to approximately 1.65 mmHg on SBP. Furthermore, the standard deviations and ICCs for SBP were significantly greater than those anticipated in the original CRHI sample size calculation. Therefore, the confidence interval did cover the expected treatment effect of −1.6 mmHg reduction. Nonetheless, taking up the intervention program only by half was the fundamental factor that led to the failure of our study to detect a possible effect.

Our results demonstrated the importance of basing public health policies on evidence of effectiveness, not only efficacy. There is no doubt about the efficacy of antihypertensive treatment among high-risk patients, and there is clear evidence that the extent of the effects increases in direct proportion to the extent to which a blood pressure reduction is achieved and the initial predicted risk of a major CVD event (19–21). However, as shown from the results of the CRHI, efficacy does not guarantee effectiveness. Other studies evaluating the treatment effects of high-risk target strategies in the prevention of cardiovascular diseases have also shown that these interventions are not effective in the general population (22–24). The development of affordable effective strategies to control the growing burden of hypertension in rural China and most other resource-poor regions of the world remains a major public health challenge (25–27). Almost certainly, this will require a two-pronged approach: first, a primary care strategy that identifies all high-risk individuals in the community and substantially reduces their risk through the combination of highly effective drug treatments and lifestyle changes (25, 28–30). Second, a population-wide strategy is needed to reduce the risk of exposure to causal factors such as high salt intake (31–33). The recent success of potassium-enriched, low-sodium salt substitutes among older adults living in elderly care facilities in China showed the promise of salt substitutes as a population-wide strategy for the prevention and control of hypertension and related diseases. Recent studies demonstrated that polypill may be useful in the prevention of cardiovascular disease by improving multiple cardiovascular risk factors at the same time, particularly for underserved populations (34, 35). With lower cost and enhanced adherence, the polypill should also fit into the needs in prevention and control of cardiovascular disease in rural China. Unfortunately, the polypill is not available on China's market yet.

There were some limitations. First, due to funding restriction and lacking existing health information system, the CRHI was forced to employee blood pressure as the study outcome to reduce the sample size and cost. Future studies should consider using clinical events as the study outcome that should better reflect the intervention effect, particularly for the intervention consisted of multiple components on different risk factors. Second, no laboratory measurements were included in our protocol for high-risk individual screening, which might reduce the precision in the estimation of the 10-year cardiovascular risk. However, the intervention was tentatively tailored for scaling up in the resource-limited healthcare settings and there is evidence that the simple, inexpensive and non-invasive non-laboratory-based risk prediction model classifies individuals almost identically to the laboratory-based model (36). Although we did not employee a calculator for the risk screening but a validated risk prediction model was used when our simple criteria of high-risk was developed (14). Third, the aspirin use in the CRHI risk management protocol looks like overused according to the recent updated guidelines (37). In fact, all high-risk individuals identified according to the CRHI criteria should have a 10-year cardiovascular risk of at least 10%. In addition, the risk management protocol excluded individuals with history of cerebral and non-cerebral hemorrhage and required to initiate aspirin use only when systolic blood pressure was below 140 mmHg. Thus, the practice should be considered in line with the current guidelines.

## Data Availability

The raw data supporting the conclusions of this article will be made available by the authors, without undue reservation.
